# Primary hyperparathyroidism with extensive brown tumours and distal humerus fracture—A case report

**DOI:** 10.1016/j.ijscr.2019.12.042

**Published:** 2020-01-16

**Authors:** Amit kumar Yadav, Eknath Pawar, Abhishek Harsoor, Prasanna kumar G S, Hitesh Rohra

**Affiliations:** Grant Medical College & Sir JJ Group of Hospitals Mumbai, India

**Keywords:** Brown tumour, fibula grafting

## Abstract

**Introduction:**

A brown tumour is benign bony lesion caused by localized, rapid osteoclastic turnover, resulting from hyperparathyroidism. In this report, we present a case of 25-year-old woman with multiple swelling and bone pain. Plain radiographs of the affected bones revealed multiple erosive bone tumours, which were diagnosed as brown tumours associated with primary hyperparathyroidism.

**Case report:**

A 25 year old female presented with swelling in left elbow since 1 month along with multiple swelling in body. Patient had pain in left elbow after trivial fall. X-ray showed lytic lesion of left elbow with pathological fracture. Biopsy was suggestive of brown tumour of distal humerus.

**Discussion:**

We managed this case with reconstruction of defect with fibula grafting and plating.

**Conclusion:**

Pathological fracture in young adult should always be properly investigated. A high index of suspicion is necessary to diagnose unusual presentation of Primary Hyperparathyroidism.

## Highlights

• Brown tumor is an uncommon pathognomic sign of hyperparathyroidism.
• Brown tumour more frequently asscociated with secondary than primary hyperparathyroidism (In our case it was associated with primary hyperparathyroidism).
• Distal humerus a rare site of brown tumour (Brown tumours associated with primary hyperparathyroidism usually affect the maxilla, pelvis and ribs other than long bones).

## 1 Introduction

A brown tumour is a, benign bony lesion caused by rapid osteoclastic turnover of bone, resulting from effects of Parathyroid hormone. Brown tumours are one of the most pathognomonic signs of primary hyperparathyroidism; however, they are rarely seen in clinical practice. Brown tumors have been reported in 1.5%–4.5% of patients with primary or secondary hyperparathyroidism [1].

In Primary hyperparathyroidism excess parathyroid hormone is secreted from parathyroid glands. The increased secretion of parathyroid hormone usually affects calcium and phosphate levels, resulting in hypercalcemia and hypophosphatemia. Approximately 80% of patients with primary hyperparathyroidism are asymptomatic, these patients are often identified following screening of calcium levels during other investigations [2]. The remaining 20% of patients often present with recurrent nephrolithiasis, osteoporosis, and psychiatric symptoms [3].

Here, we report a case of extensive brown tumors and pathological fractures caused by a parathyroid adenoma in a 25-year-old woman. This work has been reported in line with the SCARE 2018 criteria [4].

## 2 Case report

A 25 Year old woman had a trivial trauma 1 month back presented with pain and restriction of range of motion of left elbow. Patient also had swelling in left clavicle and left proximal tibia since 6 month. On clinical examination of elbow range of motion was restricted. The swelling was 5” × 5” in size, situated anteromedially just above the elbow joint. It was well-defined and bony hard in consistency. There were no signs of any neurovascular involvement. Regional lymph nodes were not palpable. She did not complain of any neuropsychiatric or gastrointestinal symptoms. She also did not have another past medical history including urinary stones.

**Fig. 1:**
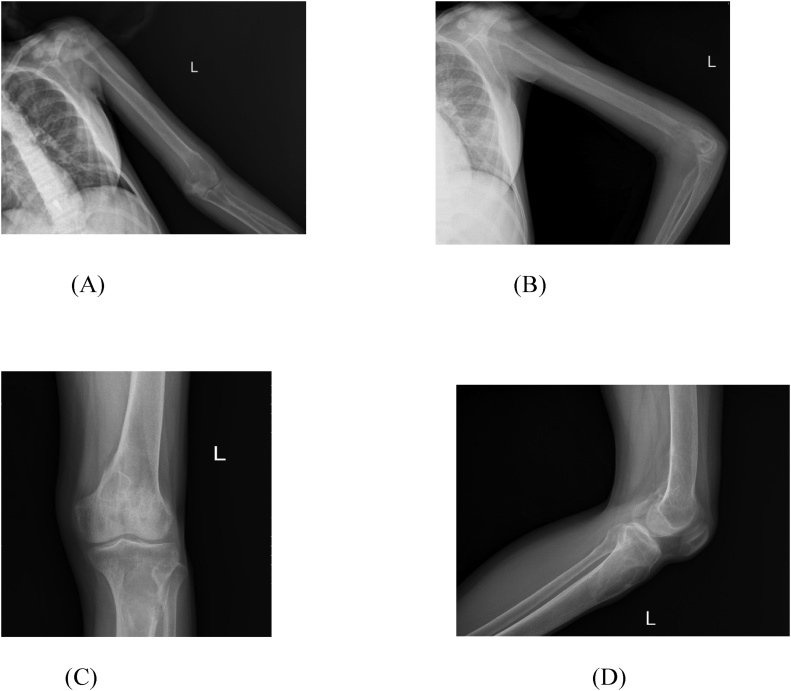
(A) Humerus AP view. (B) Humerus Lateral view. (C) Left proximal tibia AP view. (D) Left proximal tibia Lateral view.

Plain digital radiographs of the affected bones (left proximal tibia, left clavicle and left distal humerus) revealed multiple bony osteolytic expansile lesions with cortical thinning, diffuse osteoporosis, and sub periosteal bone resorption compatible with brown tumours (Fig. 1).

Initial laboratory tests indicate elevated serum calcium levels of 10.6 mg/dL, ionized calcium levels of 6.06 mg/dL, and low phosphorus levels of 0.9 mg/dL. Serum alkaline phosphatase levels were elevated at 1,802 IU/L, and serum intact PTH levels were markedly increased to 532.4 pg/mL. Twenty-four-hour urinary calcium was also elevated at 335.7 mg/day. Further laboratory tests include serum PTH-related peptide, and creatinine which were within normal limits. A parathyroid imaging work-up with neck ultrasonography and a parathyroid scan (technetium-99m sestamibi) revealed an approximately 2 cm mass in the inferior aspect of the left thyroid gland, suggestive of a solitary parathyroid adenoma. Diffuse uptake noted in region of medial end of left clavicle was suggestive of brown tumour. MRI of left elbow showed osteolytic lesion in distal humerus which was hyperintense on all the sequences. It was a well-defined lesion, and was extending into the soft tissue through a break in the medial cortex. Additional evaluation for hyperparathyroidism associated conditions was also performed.

Plain digital radiograph of the affected left distal humerus and left proximal tibia revealed bony osteolytic expansile lesions with cortical thinning, diffuse osteoporosis and sub periosteal bone resorption with definite evidence of pathological fractures in distal humerus. These findings were thought to be compatible with brown tumors of hyperparathyroidism.

**Fig. 2:**
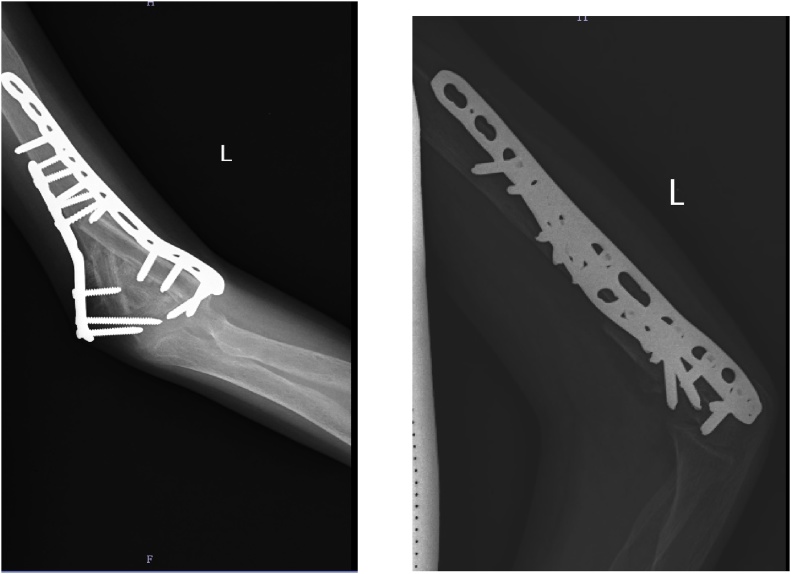
Postoperative picture showing bicolumnar plating and fibula grafting.

Pathological fracture was approached posteriorly with triceps elevating approach. The tumour was brown in appearance and was soft in consistency. Ulnar nerve was isolated and protected. Reconstruction of medial and lateral epicondyle done with fibula grafting and bicolumnar plating (Fig. 2). Anterior transposition of ulnar nerve was done. The biopsy report of the tumour lesion was identified and it showed multiple giant cells and some fibroblasts suggestive of a brown tumour or giant-cell tumour. Following management of fracture, left sided Para-thyroidectomy was performed.

Postoperatively, the patient was immobilized for 4 weeks and calcium carbonate supplementation was given.

## 3 Discussion

Primary hyperparathyroidism is a disorder in which excess Parathyroid hormone is secreted from one of the parathyroid gland. Primary hyperparathyroidism is more common in woman than man, and peak incidence occurs in 6th to 7th decade of life [5].

Brown tumour is a benign lesion that results from bony resorption due to excessive osteoclastic activity. Brown tumour is an uncommon pathognomic sign of hyperparathyroidism. Most of the reports presented a solitary lesion, generally localized to the facial bones [6,7] and more frequently associated with secondary hyperparathyroidism than primary hyperparathyroidism [8,9].

In our case the patient presented with brown tumours involving multiple bones and pathological fracture of distal humerus. Brown tumours associated with primary hyperparathyroidism usually affect the maxilla, pelvis and ribs other than long bones [10,11]. In our case, we have considered giant tumour as differential diagnosis. Peak incidence of giant-cell tumour were in the third decade of life, and usually involved the distal part of bone. In our case, a biochemical parameter and parathyroid imaging confirms the diagnosis of primary hyperparathyroidism.

## 4 Conclusion

Pathological fracture in young adult should always be investigated. A high index of suspicion is necessary to diagnose unusual presentation of primary hyperparathyroidism. Giant cell tumour should always be considered as differential diagnosis in young women.

## Source of funding

None.

## Ethical approval

This is case report study, no ethical approval were needed. In the other hand, all patient had been informed and gave their consent regarding this publication.

## Consent

Written informed consent was obtained from the patient for publication of this case report and accompanying images. A copy of the written consent is available for review by the Editor-in-Chief of this journal on request.

## Author contribution

Dr Eknath Pawar — contributed in performing the surgical procedure.

Dr Amit kumar yadav — Evaluation and post-operative management of the case along with surgical assistance, writing the paper.

Dr Abhishek harsoor — contributed in surgical assistance.

Dr Prasanna kumar G S — contributed in writing the paper.

Dr Hitesh rohra — contributed in data collection.

## Registration of research studies

This is a case report. Hence, it is not registered in the clinical trial registry.

## Guarantor

Dr Amit kumar Yadav.

## Provenance and peer review

Not commissioned externally peer reviewed.

## Declaration of Competing Interest

None.
